# Epicardial adipose tissue as an independent predictor of long-term outcome in patients with severe aortic stenosis undergoing transcatheter aortic valve replacement

**DOI:** 10.1007/s00392-024-02387-5

**Published:** 2024-02-07

**Authors:** Alexander Schulz, Bo E. Beuthner, Zoé M. Böttiger, Svante S. Gersch, Torben Lange, Judith Gronwald, Ruben Evertz, Sören J. Backhaus, Johannes T. Kowallick, Gerd Hasenfuß, Andreas Schuster

**Affiliations:** 1 Department of Cardiology and Pneumology, University Medical Center Göttingen, Georg-August University, Robert-Koch-Str. 40, 37099 Göttingen, Germany; 2 German Center for Cardiovascular Research (DZHK), Partner Site Göttingen, Göttingen, Germany; 3 School of Biomedical Engineering and Imaging Sciences, King’s College London, London, UK; 4 Institute for Diagnostic and Interventional Radiology, University Medical Center Göttingen, Georg-August University, Göttingen, Germany

**Keywords:** Epicardial adipose tissue, Transcatheter aortic valve replacement, Risk prediction, Computed tomography, Long-term outcome

## Abstract

**Background:**

Accurate risk stratification is important to improve patient selection and outcome of patients with severe aortic stenosis (AS) undergoing transcatheter aortic valve replacement (TAVR). As epicardial adipose tissue (EAT) is discussed to be involved in cardiovascular disease, it could be useful as a marker of poor prognosis in patients with severe AS undergoing TAVR.

**Methods:**

A total of 416 patients diagnosed with severe AS by transthoracic echocardiography were assigned for TAVR and enrolled for systematic assessment. Patients underwent clinical surveys and 5-year long-term follow-up, with all-cause mortality as the primary endpoint. EAT volume was quantified on pre-TAVR planning CTs. Patients were retrospectively dichotomized at the median of 74 cm^3^ of EAT into groups with low EAT and high EAT volumes. Mortality rates were compared using Kaplan-Meyer plots and uni- and multivariable cox regression analyses.

**Results:**

A total number of 341 of 416 patients (median age 80.9 years, 45% female) were included in the final analysis. Patients with high EAT volumes had similar short-term outcome (*p* = 0.794) but significantly worse long-term prognosis (*p* = 0.023) compared to patients with low EAT volumes. Increased EAT volumes were associated with worse long-term outcome (HR1.59; *p* = 0.031) independently from concomitant cardiovascular risk factors, general type of AS, and functional echocardiography parameters of AS severity (HR1.69; *p* = 0.013).

**Conclusion:**

Increased EAT volume is an independent predictor of all-cause mortality in patients with severe AS undergoing TAVR. It can be easily obtained from pre-TAVR planning CTs and may thus qualify as a novel marker to improve prognostication and management of patient with severe AS.

**Trial registration:**

DRKS, DRKS00024479.

**Graphical abstract:**

AS, aortic stenosis; TAVR, transcatheter aortic valve replacement; EAT, epicardial adipose tissue

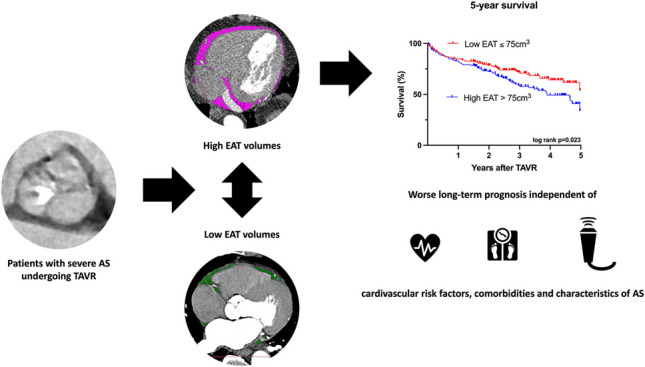

## Introduction

Severe aortic stenosis (AS) is an emerging problem in modern-day aging populations with severe affection of patients’ quality of life and overall survival [1, 2]. Within the past years, transcatheter aortic valve replacement (TAVR) has become an established and safe option for valve replacement [3, 4].

Since TAVR was introduced, numerous parameters including velocity and pressure gradients or calcification were demonstrated to impact on outcome [4–6]. This imaging-based risk prediction allows for advanced patient selection and improves procedural safety [7, 8]. Even though parameters for short- and intermediate-term outcome prognostication have been established, [9] data for the prediction of long-term outcome are scarce [10].

With the development of more advanced imaging markers, it is now possible to enhance pathophysiological understanding of different outcomes in TAVR patients [6, 11–13].

Epicardial adipose tissue (EAT) is considered a promising marker in cardiovascular disease and current research in cardiology [14]. EAT has paracrine and endocrine function and mediates proinflammatory and oxidative stimuli which nurture cardiovascular disease [15, 16]. As a result, it has been outlined as an important risk factor in various diseases such as coronary artery disease, heart failure, and heart rhythm disorders [17–19].

In patients with severe AS undergoing TAVR, EAT was not only associated with short- and medium-term outcome [12] but also with increased rates of post-interventional pacemaker implantation [20], which makes EAT an important factor within this patient group.

However, little is known about the association of EAT and outcome in different subtypes of AS [6] and long-term prognosis in general. As EAT has proven its potential as an outcome predictor, we set out to independently validate previous findings within a well-characterized cohort. Furthermore, we hypothesized that EAT impacts long-term outcome of patients with severe AS undergoing TAVR independently from subtype and other risk factors.

## Methods

### Patient cohort

A total number of 1395 patients with symptomatic severe AS were scheduled for TAVR between January 2017 and March 2022 at the Heart Center of the University Medical Center of Göttingen. After screening for inclusion and exclusion criteria as previously described [21], a total number of 416 patients were enrolled in this study. All patients were diagnosed according to current guideline recommendations [4] using echocardiography during clinical routine on either a Philips ie33 or a Philips Epic7 (Phillips Healthcare, Eindhoven, Netherlands) system. Images were stored in a picture archiving and communication system and retrospectively reevaluated according to current recommendations by a single observer using Q Station 3.8.5 (Philips Healthcare, Eindhoven, Netherlands) [22]. Patients were assigned to distinct groups of AS (e.g., low-flow low-gradient, high-flow high-gradient) following guideline recommendations [23]. This includes the high-flow high-gradient stenosis with preserved (maximum velocity (Vmax) ≥ 4 m/s, mean pressure gradient (PG) ≥ 40 mmHg, stroke volume index (SVI) > 35 ml/m^2^, ejection fraction (EF) ≥ 50%; group I) and with reduced ejection fraction (Vmax ≥ 4 m/s, mean PG ≥ 40 mmHg, SVI > 35 ml/m^2^, EF < 50%; group II) as well as the classical low-flow low-gradient (Vmax < 4 m/s, mean PG < 40 mmHg, SVI < 35 ml/m^2^, EF < 50%; group III) and paradoxical low-flow low-gradient stenosis (Vmax < 4 m/s, mean PG < 40 mmHg, SVI < 35 ml/m^2^, EF ≥ 50%; group IV).

Clinical parameters including NYHA class, cardiovascular risk factors, medical history, and laboratory markers were systematically assessed before the TAVR procedure. Furthermore, a record of preexisting heart rhythm disorders, including left bundle branch block; atrial fibrillation; and atrioventricular conduction disorders was documented. The last systematic follow-up assessment was performed in 05/2022 via registry office inquiry. In case of a patient’s death, detailed medical records were obtained from primary care physicians or relatives. The major clinical endpoint was defined as all-cause mortality. Moreover, the presence of a new-onset persistent left bundle branch block, atrio-ventricular block (grade II or higher), and the need of a pacemaker implantation post TAVR were documented. All patients gave written informed consent before participation. The study was approved by the local ethics committee and conducted according to the principles of the Helsinki Declaration.

### Computed tomography imaging protocol

Prior to TAVR procedure, patients underwent a standardized imaging protocol for the evaluation of the access path and the device landing zone as recommended by current expert consensus [24]. CT scans were performed on dual-source multi-detector scanners, either a Siemens MAGNETOM Flash CT (Siemens Healthcare, Erlangen, Germany) or a Siemens MAGNETOM Force CT (Siemens Healthcare, Erlangen, Germany) with prospective ECG triggering [25]. Angiography was performed using a high-pitch spiral acquisition mode with bolus tracking in the descending aorta after injection of 80 ml of contrast agent bolus (Imeron 350, Bracco Imaging, Konstanz, Germany) at a flow rate of 4 ml/s, followed by a 40 ml saline chaser with the same flow rate. Scan parameters were set as follows: 2 × 192 × 0.6 mm collimation, 250-ms rotation time, pitch of 3.2, and automated tube current adaption. A dedicated small field of view data set around the heart with medium soft convolution kernel (Siemens Bv36) and a maximum of 1.0-mm slice thickness was reconstructed for further analysis.

### Post-processing measurements in CT and calculation of epicardial adipose tissue

**Fig. 1:**
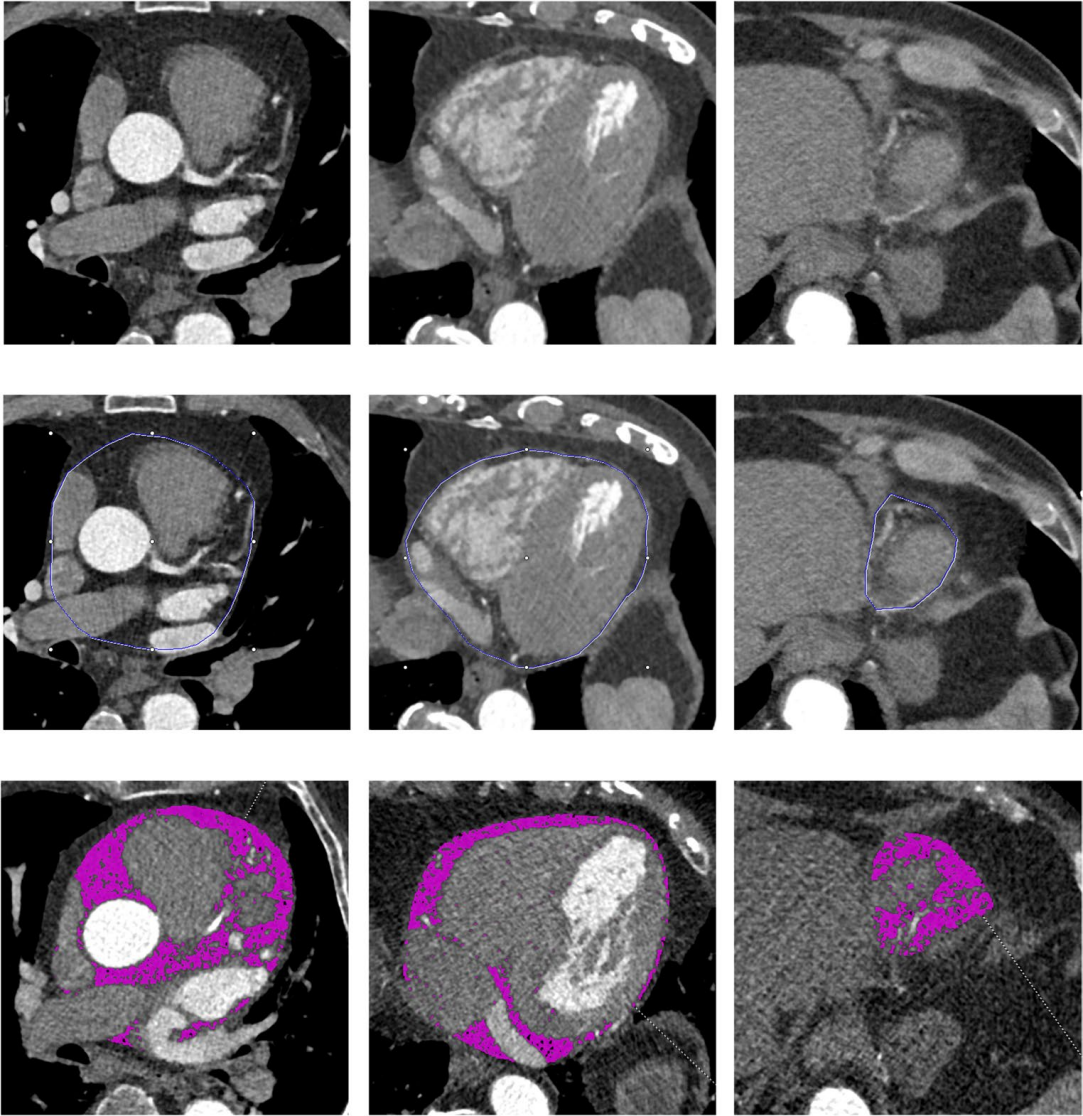
Quantification of epicardial adipose tissue using computed tomography. The images show the quantification of epicardial adipose tissue in a basal (left column), midventricular (middle column), and apical (right column) slice position. The top row shows the corresponding cardiac slices. The middle row illustrates the contouring of the pericardium. The bottom row demonstrates the volumetric quantification of all voxels within a range of − 190 to − 30 HU to calculate the epicardial adipose tissue

Post processing was performed on a dedicated commercially available software (Syngo.via, Siemens Healthcare, Erlangen, Germany). EAT was outlined by manually contouring the pericardium between the bifurcation of the main pulmonary artery (“cranial border”) and the last visible part of the pericardium (“caudal border”). Afterwards, a three-dimensional volume of interest was computed by interpolation that allowed all further analyses. Using automated thresholds of − 190 to − 30 HU, EAT was visualized (see Fig. 1). Manual corrections were performed if necessary and total volumes of EAT in cubic centimeters were documented. Intra-observer variability was calculated for 50 randomly selected patients. Inter-observer variability was calculated for 20 randomly selected patients, who were remeasured by a second experienced observer who was blinded to the previous results.

For calculation of the calcium score, an individual threshold of 550 HU was defined to distinguish between contrast agent and calcifications as recommended [6, 26, 27]. Calcium scoring was performed within the valve leaflets, aortic annulus, or aortic wall up to the sinotubular junction.

### TAVR procedure

After evaluation in the interdisciplinary heart-team, TAVR was performed at the University Medical Center Göttingen, which is certified by the German Society of Cardiology.

Most patients received an Edwards Sapien 3™ Valve (61.4%), followed by a Medtronic Evolut™ Pro (25.5%), and Medtronic Evolut™ R (9.8%). A minority of 3.2% received other valves.

### Statistical analysis

All statistical analyses were performed using SPSS statistics Version 28.0.0.1 (IBM, Armonk, NY, USA) and GraphPad Prism 9 (GraphPad Software, CA, USA). Normal distribution was tested using the Shapiro–Wilk test. Categorical variables are displayed as frequencies with corresponding percentages and were compared using the chi-square test. Continuous variables are shown as median with corresponding interquartile ranges (IQR) and were compared using the nonparametric Mann–Whitney *U* test or Kruskal–Wallis test.

Inter- and intra-observer variability was quantified by measuring the bias of two experienced observers as well as by the calculation of the intra-class correlation (ICC). The ICC was reported with the corresponding 95% confidence interval. Results of the ICC were interpreted as suggested by Bobak et al [28]. Furthermore, a coefficient of variation was calculated as $\mathrm{CoV}\hspace{0.17em}=\hspace{0.17em}\frac{\mathrm{standard}\;\mathrm{deviation}\;\mathrm{of}\;\mathrm{differences}}{\mathrm{mean}\;\mathrm{of}\;\mathrm{differences}}$.

After dichotomization at the median, survival analyses were performed and illustrated by Kaplan–Meier plots. Mortality was compared using the log-rank test. Proportional hazard assumption was verified by statistically testing the variables’ interaction with time by comparing the time-dependent covariable with the original covariable. To obtain hazard ratios (HR) of individual parameters, a logistic univariable cox regression analysis was performed. Parameters with a significant association (*p* < 0.05) with the primary endpoint were included for further analysis in multivariable cox regression analyses after adjustment for age, sex, and BMI. To avoid overfitting, two different models were tested, a first one including cardiovascular risk factors and the type of aortic stenosis, and a second one including specific parameters characterizing the subtypes of aortic stenosis. Significance levels below 0.05 are considered as statistically significant.

## Results

### Patient cohort

**Fig. 2:**
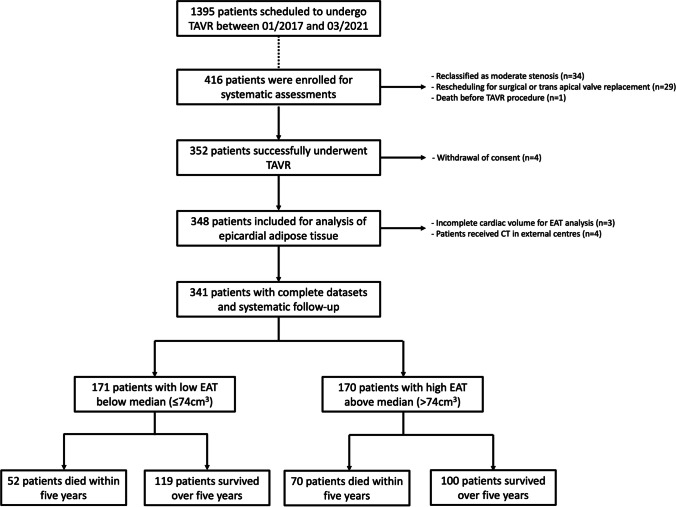
Study flowchart. TAVR, transfemoral aortic valve replacement; EAT, epicardial adipose tissue; CT, computer tomography

From the initially recruited 416 patients, 29 had to be excluded due to rescheduling for surgical or trans-apical valve replacement. Thirty-four patients had to be excluded as they were retrospectively reclassified as moderate AS before the procedure. Four patients withdrew their consent for participation before the procedure. One patient died before intervention due to infective endocarditis of the aortic valve. In three patients, the caudal end of the heart was not included within the cardiac acquisition, and 4 patients had their cardiac CT performed at external centers prior to intervention and had to be excluded from further analysis. After retrospective dichotomization at the median of the total EAT volume in the cohort, 171 patients were allocated to the low EAT volume group (≤ 74 cm^3^) and 170 patients were assigned to the high EAT volume group (> 74 cm^3^). An overview is displayed in Fig. 2.

### Reproducibility

EAT volumes as calculated by CT had excellent reproducibility with an ICC of 0.978 (0.960–0.987; *p* < 0.001) and a CoV of 9.3%. Inter-observer variability was comparably good with an ICC of 0.994 (0.984–0.997; *p* < 0.001) and a CoV of 5.2%.

### Baseline characteristics

The median age of the cohort was 80.9 (76.3–83.9) years with no differences between the high and the low EAT volume group. One hundred fifty-two (45%) patients were female, with more female patients in the low EAT volume group compared to the high EAT volume group (89 vs. 63; *p* = 0.005). Patients in the high EAT volume group had a higher BMI (28.8 vs. 25.0; *p* < 0.001), higher NYHA classes (*p* = 0.016), and a higher prevalence of diabetes mellitus type II (47 vs. 73; *p* = 0.003). Other comorbidities and cardiovascular risk factors were equally distributed between both groups (compare Table 1).

**Table 1:** Baseline characteristics of patients with high and low EAT

Parameter	Low EAT (*n* = 171)	High EAT (*n* = 170)	*p*-value
Patient characteristics and laboratory markers			
Age (years)	80.9 (77.5; 83.9)	80.5 (75.9; 84.0)	0.446
Sex: female/male (*n*; %)	89 (52)/82 (48)	63 (37)/107 (63)	0.005*
BMI (kg/m^2^)	25.0 (23.2; 32.3)	28.8 (25.9; 32.3)	< 0.001*
NYHA class: I/II/III/IV (*n*; %)	8 (4)/68 (40)/85 (50)/10 (6)	6 (3)/42 (25)/105 (62)/17 (10)	0.016*
NT-proBNP (ng/l)	1940.0 (597.6; 4610.2)	1417.7 (719.0; 3702.0)	0.617
Kreatinin (mg/dl)	1.04 (0.82; 1.35)	1.10 (0.89; 1.39)	0.171
EuroScore II	3.5 (1.8; 6.5)	3.2 (2.1; 5.8)	0.929
Medical history			
Diabetes (*n*; %)	47 (27)	73 (43)	0.003*
Arterial hypertension (*n*; %)	145 (85)	155 (91)	0.070
Hyperlipoproteinemia (*n*; %)	106 (62)	114 (67)	0.292
Increased family risk (*n*; %)	21 (12)	16 (9)	0.615
CAD (*n*; %)	102 (60)	112 (66)	0.234
Past myocardial infarction (*n*; %)	22 (13)	22 (13)	0.983
Cardiomyopathy (*n*; %)	9 (5)	6 (4)	0.435
Atrial fibrillation/flutter (*n*; %)	65 (38)	80 (47)	0.091
Implanted ICD or PM (*n*; %)	30 (18)	26 (15)	0.575
PAD (*n*; %)	11 (6)	25 (15)	0.013*
Past stroke (*n*; %)	24 (14)	23 (14)	0.875
Echocardiography indices			
Ejection fraction (%)	54.2 (44.0; 60.0)	54.0 (42.5; 59.2)	0.498
Stroke volume index (ml/m^2^)	33.5 (28.6; 40.0)	33.0 (27.1; 40.0)	0.484
Maximum velocity (m/s)	4.1 (3.4; 4.4)	4.1 (3.6; 4.5)	0.476
Mean pressure gradient (mmHg)	38.0 (27.0; 49.0)	39.5 (30.0; 47.0)	0.752
Aortic opening area (cm^2^)	0.65 (0.5; 0.8)	0.70 (0.6; 0.8)	0.011*
Type of stenosis: I/II/III/IV (*n*; %)	80 (47)/21 (13)/35 (20)/35 (20)	81 (48)/31 (18)/30 (18)/28 (16)	0.378
Measurements in computed tomography			
EAT (cm^3^)	52.8 (44.0; 62.7)	103.4 (87.1; 128.8)	< 0.001*
Calcium scoring	573.5 (334.3; 875.1)	695.1 (447.6; 980.0)	0.022*

Analysis of baseline characteristics after dichotomization at the median of EAT at 74 cm^3^. The asterisk indicates statistical significance. *EAT*, epicardial adipose tissue; *BMI*, body mass index; *CAD*, coronary artery disease; *ICD*, implantable cardioverter defibrillator; *PM*, pacemaker; *PAD*, peripheral artery disease

The baseline characteristics and echocardiographic indices of patients with different types of AS are summarized in Table 2. For the EAT volume, no differences between the individual groups could be found (I 75.0 vs. II 86.3 vs. III 71.1 vs. IV 68.3 cm^3^; *p* = 0.822).

**Table 2:** Baseline characteristics of patients with different types of aortic stenosis

Parameter	AS group I (*n* = 161)	AS group II (*n* = 52)	AS group III (*n* = 65)	AS group IV (*n* = 63)	*p*-value
Patient characteristics and laboratory markers					
Age (years)	79.9 (75.6; 83.5)	80.5 (75.7; 86.5)	80.8 (77.0; 83.2)	82.9 (79.9; 85.3)	0.257
Sex: female/male (*n*; %)	82 (51)/79 (49)	18 (35)/34 (65)	16 (25)/49 (75)	36 (57)/27 (43)	< 0.001*
BMI (kg/m^2^)	27.4 (24.4; 30.6)	26.5 (24.8; 31.7)	26.3 (23.0; 30.5)	26.5 (24.2; 29.8)	0.431
NYHA class: I/II/III/IV (*n*; %)	10 (6)/62 (39)/85 (53)/4 (2)	0/17 (33)/26 (50)/9 (17)	0/14 (22)/42 (64)/9 (14)	4 (6)/17 (27)/37 (69)/5 (8)	< 0.001*
NT-proBNP (ng/l)	871.4 (372.1; 2048.0)	4887.6 (2837.0; 10,982.0)	3918.3 (1398.0; 11,422.8)	1438.3 (881.8; 3268.0)	< 0.001*
Kreatinin (mg/dl)	0.99 (0.80; 1.27)	1.02 (0.91; 1.45)	1.18 (0.95; 1.48)	1.13 (0.87; 1.34)	< 0.001*
EuroScore II	2.3 (1.6; 4.1)	4.0 (2.4; 8.6)	5.9 (3.1; 10.5)	3.1 (1.9; 4.7)	< 0.001*
Echocardiography indices					
Ejection fraction (%)	58.4 (54.9; 63.6)	39.4 (29.4; 44.5)	36.9 (29.3; 44.3)	55.1 (53.1; 58.5)	< 0.001*
Stroke volume index (ml/m^2^)	38.7 (32.4; 45.9)	29.9 (25.5; 37.4)	28.7 (23.1; 34.4)	29.7 (25.0; 33.0)	< 0.001*
Maximum velocity (m/s)	4.3 (4.1; 4.6)	4.3 (4.1; 4.7)	3.4 (3.0; 3.6)	3.4 (3.0; 3.6)	< 0.001*
Mean pressure gradient (mmHg)	44 (40; 52)	45 (41; 55)	25 (20; 30)	24 (21; 31)	< 0.001*
Aortic opening area (cm^2^)	0.70 (0.59; 0.80)	0.60 (0.50; 0.70)	0.70 (0.60; 0.83)	0.71 (0.60; 0.80)	< 0.001*
Measurements in computer tomography					
EAT (cm^3^)	75.0 (57.8; 101.7)	86.3 (53.5; 115.9)	71.1 (48.5; 106.0)	68.3 (53.1; 99.0)	0.822
Calcium scoring	699.7 (430.3; 928.4)	904.9 (595.7; 1222.4)	486.2 (331.2; 708.1)	420.0 (211.0; 733.4)	< 0.001*

Analysis of baseline characteristics in the different groups of aortic stenosis. Group I, high-flow high-gradient stenosis with preserved ejection fraction (EF); group II, high-flow high-gradient stenosis with reduced EF; group III, low-flow low-gradient stenosis with reduced EF; group IV, low-flow low-gradient stenosis with preserved EF. The asterisk indicates statistical significance. *AS*, aortic stenosis; *EAT*, epicardial adipose tissue; *BMI*, body mass index

### Echocardiography

**Fig. 3:**
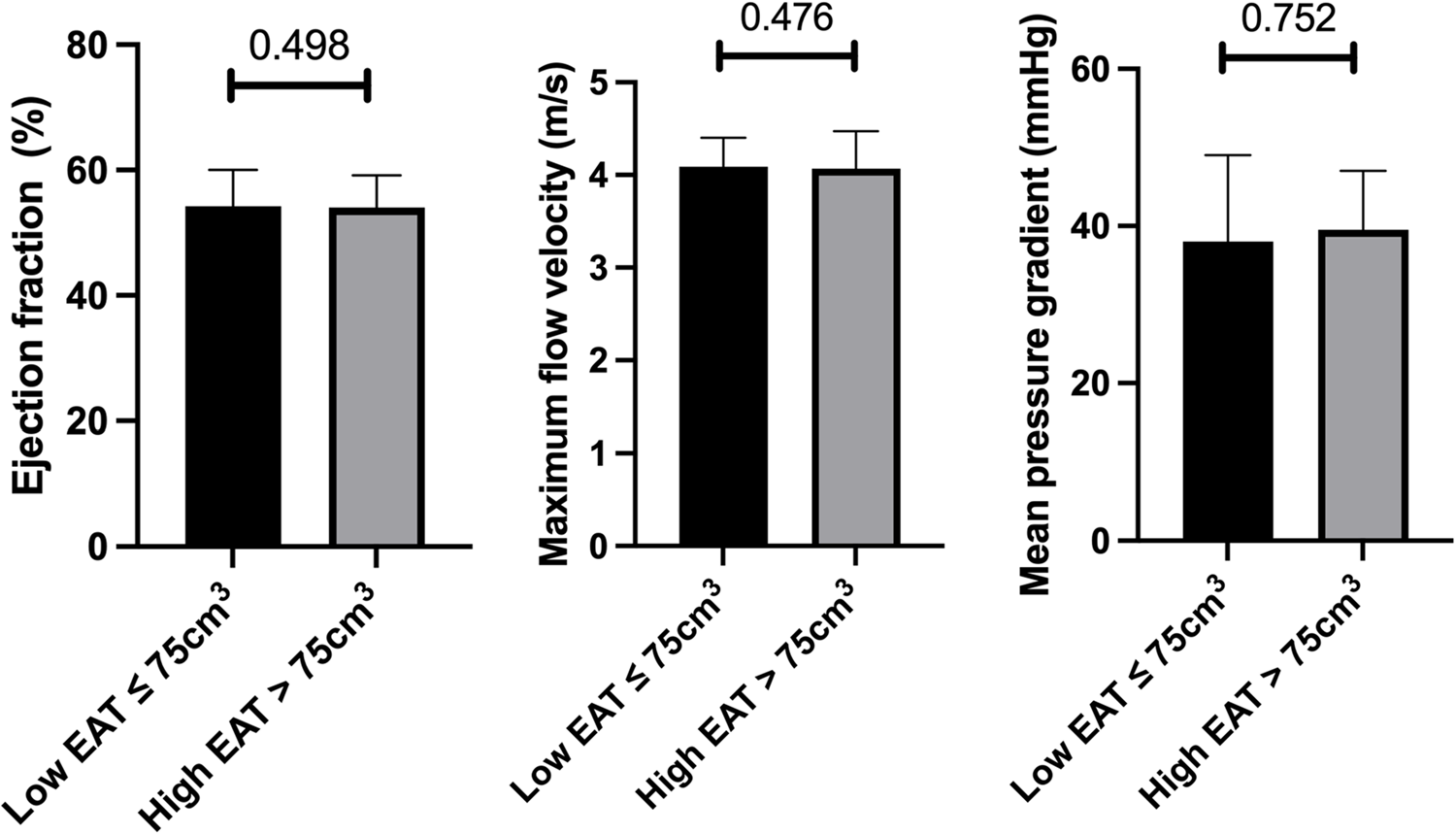
Characteristics of the aortic stenosis in patients with high and low EAT volumes. The image shows the median (interquartile range) of the ejection fraction, the maximum flow velocity, and mean pressure gradient of the aortic valve in patients with low (≤ 75 cm^3^) and high (> 75 cm^3^) epicardial adipose tissue (EAT)

Patients in the high and the low EAT volume groups had no differences between ejection fraction, mean PG, or maximum flow velocity over the aortic valve (see Fig. 3). In the group of patients with low EAT volumes, the aortic valve area was slightly smaller compared to the group of patients with high EAT volumes (0.65 vs. 0.70, 0.011) (see Table 1).

### Outcome and EAT

**Fig. 4:**
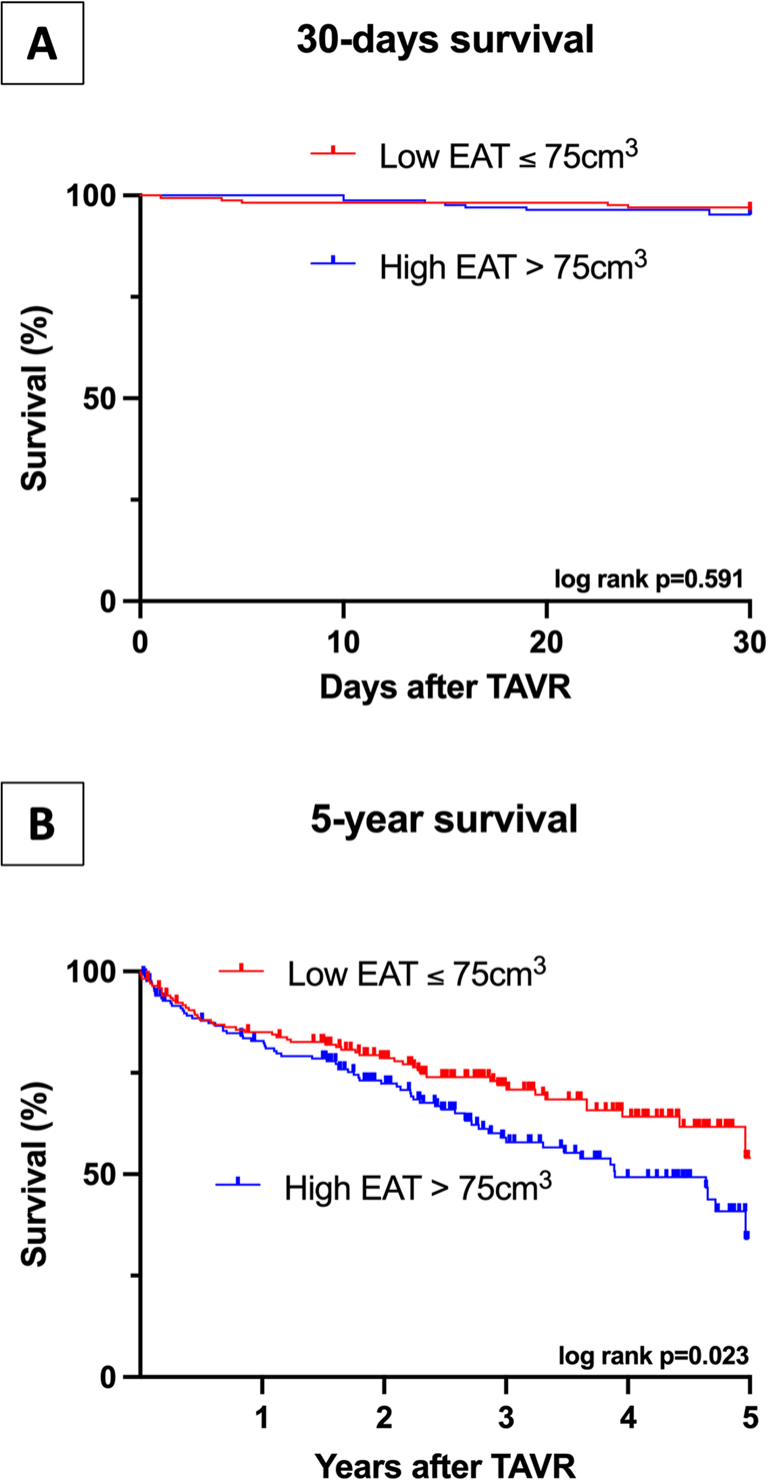
Survival after TAVR. The top graph (**A**) shows the 30-day survival after TAVR in the low (red) and high (blue) EAT volume group. The bottom graph (**B**) displays the long-term 5-year survival after TAVR in the low (red) and high (blue) EAT volume group. A *p*-value below 0.05 was considered statistically significant. EAT, epicardial adipose tissue; TAVR, transfemoral aortic valve replacement

There were no differences between short-term outcome within 30 days after TAVR (log rank *p* = 0.591) (see Fig. 4A). In contrast, patients with higher EAT volumes had a significantly higher 5-year all-cause mortality compared to patients with low EAT volumes (log rank *p* = 0.023) (see Fig. 4B). As shown in Table 3, there were no differences regarding the occurrence of heart rhythm disorders after TAVR between the high and low EAT volume group. There was only a statistical trend towards a higher rate of pacemaker implantations post-TAVR in patients with higher EAT volume (15 vs. 25, *p* = 0.088).

**Table 3:** Event rates in the low and high EAT volume group

Event	Low EAT (*n* = 171)	High EAT (*n* = 170)	*p*-value
30-day all-cause mortality (*n* (%))	8 (4)	7 (4)	0.855
5-year all-cause mortality (*n* (%))	52 (30)	70 (41)	0.038*
New LBBB (*n* (%))	22 (13)	28 (16)	0.358
2nd degree AV block or above (*n* (%))	18 (11)	19 (11)	0.862
New pacemaker (*n* (%))	15 (9)	25 (16)	0.088
New onset atrial fibrillation/flutter (*n* (%))	12 (7)	9 (5)	0.536
Other relevant heart rhythm disorders (*n* (%))	6 (4)	12 (7)	0.132

Events in the low and high EAT volume group divided by the median. The median value of calculated EAT volumes was 74 cm^3^. The single asterisk (*) indicates statistical significance. *EAT*, epicardial adipose tissue; *LBBB*, left bundle branch block; *AV*, atrio-ventricular

In univariable cox regression analysis, presence of diabetes mellitus (HR 1.63, *p* = 0.007), a positive family history of cardiovascular events (HR 0.48; *p* = 0.048), peripheral artery disease (HR 2.0; *p* = 0.003), and the type of AS (HR 1.37; *p* < 0.001) were associated with increased mortality after TAVR (see Table 4).

**Table 4:** Uni- and multivariable analysis of hazard ratios of cardiovascular risk factors and comorbidities

Parameter	Univariable HR	*p*-value	Multivariable HR**	*p*-value
Diabetes	1.629 (1.140–2.328)	0.007*	1.708 (1.142–2.554)	0.009*
Arterial hypertension	1.236 (0.665–2.298)	0.503		
Hyperlipoproteinemia	0.953 (0.659–1.379)	0.798		
Increased family risk	0.484 (0.236–0.993)	0.048*	0.575 (0.277–1.191)	0.136
CAD	1.150 (0.789–1.674)	0.467		
PAD	2.006 (1.264–3.185)	0.003*	1.295 (0.773–2.169)	0.325
Past stroke	1.408 (0.894–2.217)	0.140		
Type of stenosis	1.372 (1.185–1.588)	< 0.001*	1.353 (1.163–1.575)	< 0.001*
EAT above median	1.483 (1.035–2.123)	0.032*	1.585 (1.044–2.407)	0.031*

Univariable and multivariable analysis of the hazard ratios of associated parameters. The median of EAT was defined at 74 cm^3^. The single asterisk (*) indicates statistical significance. *EAT*, epicardial adipose tissue; *BMI*, body mass index; *CAD*, coronary artery disease; *PAD*, peripheral artery disease. **Corrected for age, BMI, and sex

Furthermore, EAT volumes above the median predicted a poorer 5-year long-term outcome with a HR of 1.48 (1.04–2.12; *p* = 0.032). In multivariable cox regression analysis, after adjustment for age, sex, and BMI, increased EAT volumes above the median still showed a significant association with mortality after TAVR (HR 1.59 1.04–2.41; *p* = 0.031) independently from cardiovascular risk factors, comorbidities, and AS subtype (see Table 4). In addition, increased EAT volume above the median was the only parameter remaining associated with increased mortality after TAVR in multivariable cox regression analysis including specific parameters characterizing the aortic stenosis like the SVI, Vmax, mean PG, or the calcium scoring (HR 1.69 1.12–2.54; *p* = 0.013) (Table 5).

**Table 5:** Uni- and multivariable analysis of hazard ratios of parameters characterizing the aortic stenosis

Parameter	Univariable HR	*p*-value	Multivariable HR**	*p*-value
Ejection fraction	0.987 (0.974–1.000)	0.058		
Stroke volume index	0.972 (0.953–0.992)	0.005*	0.990 (0.968; 1.013)	0.406
Maximum velocity	0.667 (0.515–0.864)	0.002*	1.185 (0.492; 2.853)	0.705
Mean pressure gradient	0.979 (0.966–0.992)	0.001*	0.971 (0.929; 1.014)	0.182
Aortic valve opening area	1.634 (0.534–5.003)	0.390		
Calcium scoring	1.000 (0.999–1.000)	0.097		
EAT above median	1.483 (1.035–2.123)	0.032*	1.686 (1.118; 2.542)	0.013*

Univariable and multivariable analysis of the hazard ratios of associated parameters. The median of EAT was defined at 74 cm^3^. The single asterisk (*) indicates statistical significance. *EAT*, epicardial adipose tissue; *BMI*, body mass index. **Corrected for age, BMI, and sex

## Discussion

Risk stratification in patients undergoing TAVR is important to enhance the outcome of the procedure and to improve selection of patients that would benefit from interventional therapies [29, 30]. In addition to established parameters, calculation of EAT volume using cardiac CT offers a valuable tool for improved risk stratification. While the short-term outcome did not differ between patients with high and low EAT volumes, increased EAT volumes predicted a worse long-term outcome in TAVR patients. This was independent of patients’ cardiovascular risk factors as well as the subgroup of severe AS. Interestingly, EAT volumes had no impact on cardiovascular conduction disorders after TAVR, although we observed a trend towards a higher rate of pacemaker implantations in patients with high EAT volumes.

As EAT volumes can be sufficiently assessed from pre-TAVR planning CTs, its quantification can be performed without the need for additional diagnostic procedures or the application of additional radiation.

Conventional cardiovascular risk factors were validated for risk stratification in patients with severe AS, and summarized in scores such as the EUROSCORE II to assign patients for SAVR or TAVR [31]. However, it was shown that those scores require adjustments within the population of patients scheduled for TAVR to improve outcome prediction [32–34].

An emerging parameter receiving significant attention in cardiovascular medicine is EAT providing novel insights into pathophysiology in various cardiovascular diseases [14, 35, 36].

EAT was shown to be a risk factor in atherosclerotic coronary artery disease and is independently associated with a higher risk of obstructive CAD, atrial fibrillation, and heart failure. [18, 19, 36–38] Moreover, increased EAT volumes are associated with increased rates of cardiovascular events and worse outcome independent of conventional cardiovascular risk factors even in the general population [17].

Pathophysiologically, the interconnection of EAT and cardiovascular remains a matter of debate. On the one hand, EAT is assumed to generate proinflammatory cytokines causing structural damage and promoting adverse remodeling of adjacent tissue within the myocardium and attached structures [14, 16]. Moreover, as EAT is significantly associated with increased coronary artery calcification, it is also suspected to promote atherosclerosis [39, 40]. Likewise, it could be an important inflammatory mediator contributing to aortic valve calcification in the same way [41]. In the present study, this was partly confirmed by higher aortic calcium scores in the group of patients with increased EAT volumes. However, this direct involvement of EAT in the development and progression of AS has yet to be demonstrated.

On the other hand, the causal interaction of EAT with cardiovascular diseases could rather be bidirectional. Cardiovascular diseases and risk factors like atherosclerosis, atrial fibrillation, diabetes, or obesity may cause systemic dysregulation in the first place which is then promoting the development, progression, and remodeling of EAT as a symptom-like phenomenon. This is partly paralleled by the observation of a protective role of EAT in early years of life and a consecutive remodeling of EAT at later age stages causing adverse effects on the cardiovascular system [14, 42]. The complex interaction of cardiovascular disease and EAT mutually promoting each other and leading to the observed association of EAT with pathological cardiovascular conditions will need to be further investigated in prospective studies including dedicated control groups.

Therefore, increased volumes of EAT in patients might just represent a generally sicker group of patients in more advanced stages of their disease, which is concordantly indicated by higher NYHA classes in the group of patients with higher EAT volumes.

Since EAT volumes can be easily quantified on conventional TAVR planning CTs (that are being performed in any given patient) without the need for an additional diagnostic procedure or radiation exposure, it may well be of predictive value in this patient population.

Interestingly, total CT-derived EAT volumes are very variable across different study populations ranging from a median of 71 to 90 ml in younger patient populations with cardiovascular disease [43–46] up to 127 to 130 ml in older patient populations [12, 20]. EAT volumes of the present study are rather low, despite a similar age and risk factor distribution of the current cohort compared to patients in the latter studies. While this could also be attributed to distinct patient characteristics, it is more likely that methodological differences in the evaluation of EAT volumes are accountable for the heterogeneity.

While the cranial and caudal border for EAT volume quantification was mostly the same in all studies, a large meta-analysis already reported that different Hounsfield ranges were used for EAT volume evaluation as well as some studies analyzed EAT volumes in more systolic or diastolic cardiac phases [47]. Other factors like image resolution, the method of reconstruction, and the analysis of contrast-enhanced or non-contrast scans could further influence EAT measurements. This emphasizes the need for further standardization with regard to measurement techniques before utilizing EAT volumes for risk stratification in clinical routine.

Despite potential methodological divergences, the assessment of EAT volumes was reported to be a valuable prognostic tool in cardiovascular disease in general [17, 37, 47–49]. Only a few studies investigated the impact of EAT volumes on the outcome of patients with AS undergoing TAVR. Even though total EAT volumes are different to previously investigated populations, first studies have already outlined that EAT quantification can enhance risk prediction in TAVR patients [50] and higher EAT volumes are associated with worse short-term and intermediate-term outcome [12]. While this study was now able to add implications on long-term outcome over a 5-year period, we were not able to reproduce earlier findings on the short-term outcome. On the one hand, this may be attributed to the low number of events within 30 days after TAVR. On the other hand, it could be a consequence of differing EAT volumes, patient comorbidities, or procedural differences. For example, another study reported significant associations between relevant heart rhythm disorders and higher amounts of EAT [20], which is a well-known short-term complication after TAVR. In contrast, the present study revealed similar rates of post-TAVR conduction abnormalities between both groups potentially indicating different patient selection or intra- and post-procedural management.

Furthermore, it should be noted that earlier trials reported a paradoxical effect of obesity in patients undergoing TAVR as obese patients have been observed to have a lower mid- and long-term mortality compared to patients having normal weight [51–53]. However, in the present study, patients with increased EAT volume had a concordantly increased BMI but worse outcome. While the association of increased EAT volumes and obesity was described earlier [54], the worse outcome of those patients is contradictive to the protective effect of obesity. Interestingly, in the present study, patients with both high and low EAT volumes belong to the same subgroup of overweight but non-obese patients with a BMI between 25 and 29.9 kg/m^2^. Patients in this BMI range were defined as a single population in the abovementioned studies and compared with patients in other BMI ranges. This could partly explain the smaller impact of the BMI on outcome in this study. Furthermore, as patients belonged to the same BMI range, the reported difference in mortality potentially points out to an additional value of EAT for sub-characterization in overweight patients.

While the inhomogeneity of the disease alone with different types of severe AS (e.g., low-flow low-gradient vs. high-flow high-gradient) has implications on outcome [55], this study could now add that EAT volume quantification allows an independent risk stratification in TAVR patients independent of the subgroup of severe AS. Therefore, the addition of EAT volume as an outcome predictor in TAVR patients could add a valuable, independent outcome predictor in distinct types of severe AS [56]. Still, the disparate findings on short-term outcome and conduction abnormalities in other studies endorse the need for multiparametric outcome prediction in TAVR patients and a validation in larger prospective trials.

Using EAT volume as a predictive parameter for long-term outcome assessments is especially interesting considering that TAVR is progressively used in a younger patient collective with a longer life expectancy [57]. Multiparametric CT including prognostication with EAT volumes may therefore have important implications on interventional decision-making beyond valve size and prosthesis selection.

## Limitations

Findings of this study are based on data from a single-center cohort, within a certified and specialized institution for TAVR. Mortality rates and causes might differ in multicentric studies. The rather small number of patients within this study potentially reduces the statistical accuracy and implies that some results may or may not be relevant in larger cohorts.

The calculation of EAT volumes using CT Hounsfield units is just an estimation of the true epicardial fat volume and might also include other parts of surrounding tissue especially pericardial fat tissue. The threshold of 75 cm^3^ of EAT volume might only apply to the investigated cohort and not be applicable in other patient groups. Further studies in prospective, population-based investigations are therefore required. Without a direct comparison with a matched control group, the impact of EAT might just be a surrogate for the influence of other comorbidities or life-style factors. Despite multivariable regression analyses, the influence of other given pathologies on outcome cannot be fully excluded and implications of EAT in TAVR patients must be further investigated.

## Conclusion

Patients with severe AS and higher volumes of EAT have worse long-term but not short-term outcome after TAVR. Increased amounts of EAT predict long-term mortality and potential necessity for secondary prevention independently from the type of aortic valve stenosis, its treatment with TAVR, or accompanying cardiovascular risk factors.

As EAT volumes can be easily quantified using regular TAVR planning CTs without the need for additional diagnostic tests or radiation exposure, it could qualify as an additional prognostic and cost-effective marker in TAVR patients. Its calculation could improve risk stratification and care of patients with severe AS assigned for TAVR.
